# Supplementary material for: Evidence for Naturally Produced Beauvericins Containing N-Methyl-Tyrosine in Hypocreales Fungi
Source: Toxins (Basel). 2019 Mar 26;11(3):182. doi: 10.3390/toxins11030182 (PMC6468924; doi:10.3390/toxins11030182)
Supplement: Supplementary file 1 [file toxins-11-00182-s001.pdf]

Supplementary Materials: Evidence for Naturally Produced Beauvericins Containing *N*-Methyl-Tyrosine in *Hypocreales* Fungi

Monika Urbaniak, Łukasz Stępień and Silvio Uhlig

Table 1. HILIC–ion trap mass spectrometry characteristics of the reference amino and hydroxy acids.

Amino or hydroxy acid	Retention Time (min)	Observed *m*/*z*	Ion Species
N-methyl-phenylalanine	8.0	180.1	$[M + H]^+$
N-methyl-tyrosine	10.2	196.2	$[M + H]^+$
N-methyl-valine	9.6	132.1	$[M + H]^+$
N-methyl-leucine	8.2	146.2	$[M + H]^+$
N-methyl-isoleucine	8.5	146.2	$[M + H]^+$
D-hydroxy-isovaleric acid	5.7	117.2	$[M - H]^-$

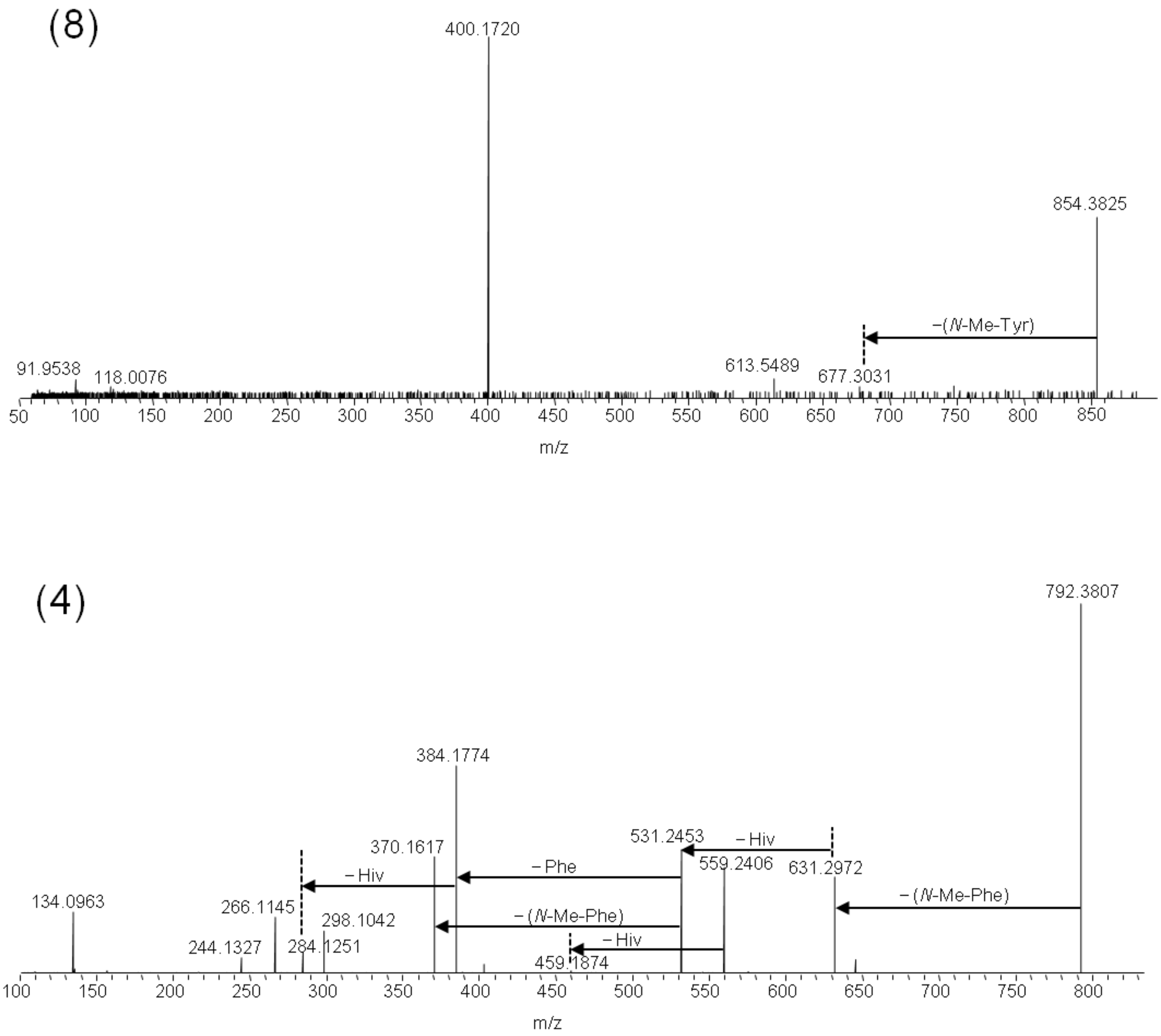

Figure 1. LC/HRMS^2 spectra of the $[M + Na]^+$ ions of beauvericin analogues: beauvericin L (**8**) containing *N*-methyl-tyrosine and beauvericin D (**4**) containing phenylalanine.

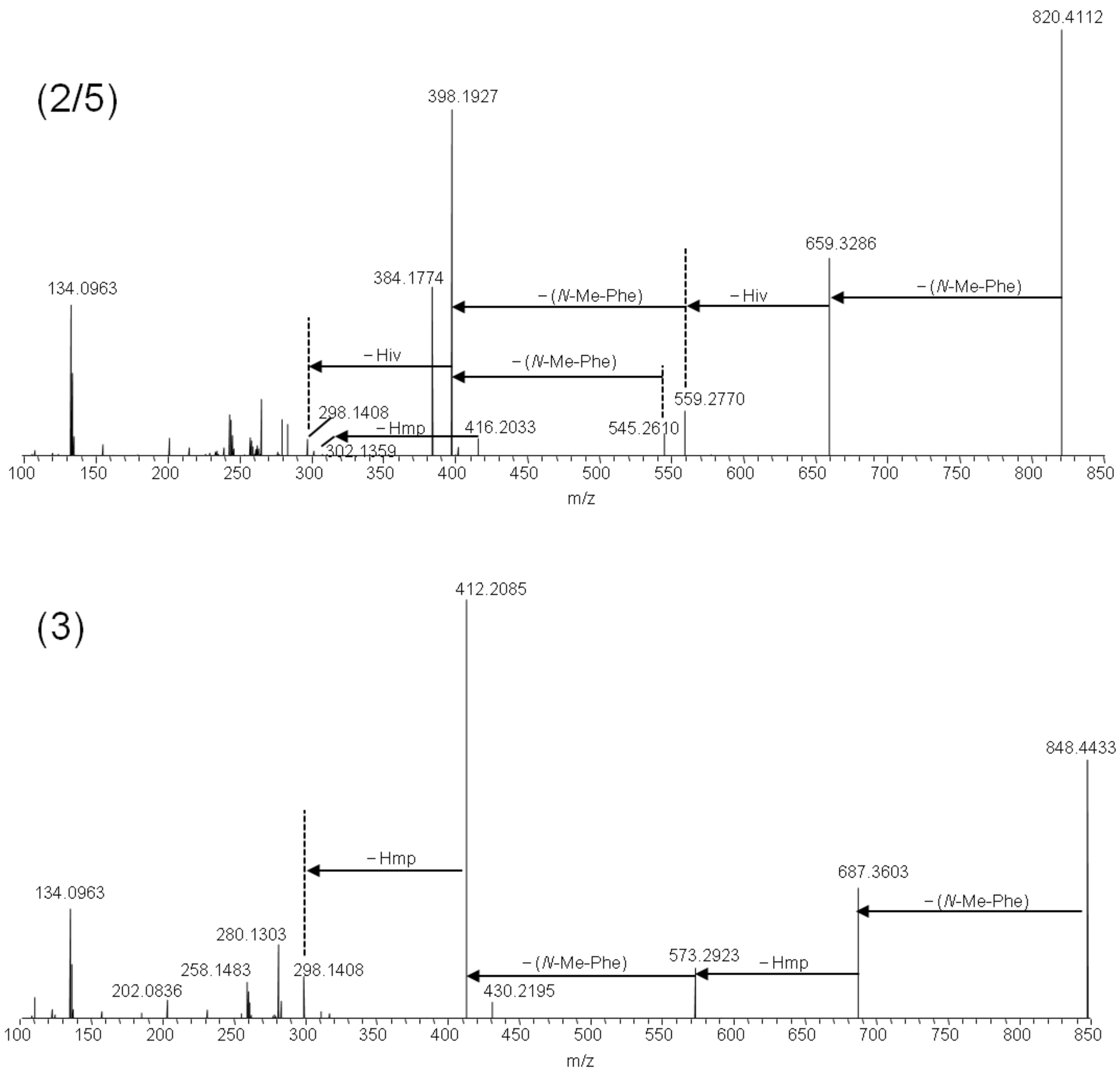

Figure 2. LC/HRMS^2 spectra of the $[M + Na]^+$ ions of beauvericin analogues containing D-Hmp (2-hydroxyisocaproic acid) group: beauvericin A/F (**2**/**5**) and beauvericin C (**3**).

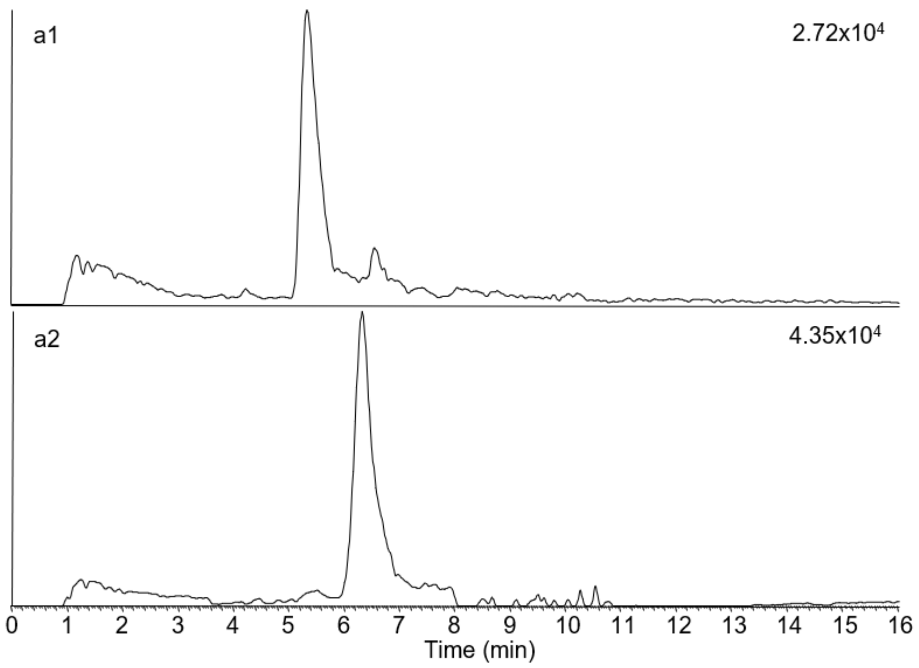

Figure 3. Ion chromatograms for [M − H]⁻ of D-Hiv (*m*/*z* 117) from HILIC–ion trap mass spectrometry. The upper trace represents a chromatogram from a pure reference standard (**a1**), while the lower trace is from a hydrolyzed depsipeptide mixture (1% P35 sample, **a2**). Individual chromatograms are scaled to the highest peak (number in the top right-hand corner of each chromatogram).
